# Design of a placebo-controlled, randomized study of the efficacy of repetitive transcranial magnetic stimulation for the treatment of chronic tinnitus

**DOI:** 10.1186/1471-244X-8-23

**Published:** 2008-04-15

**Authors:** Michael Landgrebe, Harald Binder, Michael Koller, Yvonne Eberl, Tobias Kleinjung, Peter Eichhammer, Erika Graf, Goeran Hajak, Berthold Langguth

**Affiliations:** 1 Department of Psychiatry, Psychosomatics, and Psychotherapy, University of Regensburg, Germany; 2 Center for Clinical Studies, University Hospital of Regensburg, Germany; 3 Department of Otorhinolarnygology, University Hospital of Regensburg, Germany; 4 Institute of Medical Biometry and Medical Informatics, University Medical Center Freiburg, Germany

## Abstract

**Background:**

Chronic tinnitus is a frequent condition, which can have enormous impact on patient's life and which is very difficult to treat. Accumulating data indicate that chronic tinnitus is related to dysfunctional neuronal activity in the central nervous system. Repetitive transcranial magnetic stimulation (rTMS) is a non-invasive method which allows to focally modulate neuronal activity. An increasing amount of studies demonstrate reduction of tinnitus after repeated sessions of low-frequency rTMS and indicate that rTMS might represent a new promising approach for the treatment of tinnitus. However available studies have been mono-centric and are characterized by small sample sizes. Therefore, this multi-center trial will test the efficacy of rTMS treatment in a large sample of chronic tinnitus patients.

**Methods/Design:**

This is a randomized, placebo-controlled, double-blind multi-center trial of two weeks 1 Hz rTMS-treatment in chronic tinnitus patients. Eligible patients will be randomized to either 2 weeks real or sham rTMS treatment. Main eligibility criteria: male or female individuals aged 18–70 years with chronic tinnitus (duration > 6 months), tinnitus-handicap-inventory-score ≥ 38, age-adjusted normal sensorineural hearing (i.e. not more than 5 dB below the 10% percentile of the appropriate age and gender group (DIN EN ISO 7029), conductive hearing loss ≤ 15dB. The primary endpoint is a change of tinnitus severity according to the tinnitus questionnaire of Goebel and Hiller (baseline vs. end of treatment period). A total of 138 patients are needed to detect a clinical relevant change of tinnitus severity (i.e. 5 points on the questionnaire of Goebel and Hiller; alpha = 0.05; 1-beta = 0.80). Assuming a drop-out rate of less than 5% until the primary endpoint, 150 patients have to be randomized to guarantee the target number of 138 evaluable patients. The study will be conducted by otorhinolaryngologists and psychiatrists of 7 university hospitals and 1 municipal hospital in Germany.

**Discussion:**

This study will provide important information about the efficacy of rTMS in the treatment of chronic tinnitus.

**Trial registration:**

Current Controlled Trials ISRCTN89848288

## Background

Subjective tinnitus is a frequent auditory sensation experienced in the absence of an external or internal acoustic stimulus. Tinnitus may present only sporadically or may manifest as constant ear-ringing of high intensity that entrains significant morbidity and may progress to a chronic debilitating condition. There is increasing evidence from electrophysiological and functional neuroimaging studies that tinnitus results from increased neuronal activity within the central auditory pathways [1]. Similar like in auditory hallucinations [2] the increased neuronal firing in the auditory cortex results in the perception of a phantom sound. This notion is supported by recent neuroimaging studies which point to a pathologically over activated, distributed cortical network involving the inferior colliculus [3], the thalamus [4], and the primary auditory cortex [4-7] in subjects with tinnitus. The pathophysiological relevance of this network has been demonstrated recently by transient suppression of tinnitus after high-frequency rTMS to the temporal and temporo-parietal cortex [8,9].

Low-frequency rTMS (1 Hz) is known to reduce neural activity in directly stimulated brain regions [10,11] as well as in structurally connected remote brain regions [11,12]. For these reasons low-frequency rTMS has been proposed as an innovative and causally orientated treatment strategy for pathological conditions with increased cortical activity [13]. Consequently, applied to the left temporo-parietal cortex rTMS has been repeatedly demonstrated to reduce auditory hallucinations in patients with schizophrenia [14].

In pilot studies, first evidence for a beneficial effect of magnetic resonance imaging (MRI) and positron emission tomography (PET) guided neuronavigated low-frequency rTMS has been found in patients with chronic tinnitus [15]. This finding has been confirmed by several controlled trials which all demonstrated a significant reduction of tinnitus severity after five to ten days of active rTMS as compared to sham stimulation [16-18]. Neurophysiological work could recently detect that rTMS-induced improvement in tinnitus complaints was paralleled by changes in cortical excitability, suggesting, that this beneficial therapeutic effect is associated with the induction of neuroplastic processes [19]. In line with these data, neuroimaging studies using voxel-based morphometry (VBM) demonstrated that low-frequency rTMS over auditory brain regions is able to induce profound changes in gray matter both in cortical auditory brain areas as well as in the thalamus [12]. Since neuroplastic alterations at the level of the thalamus seem to be essential in the generation of chronic tinnitus [20], low-frequency rTMS may represent a new approach for treating this disorder by inducing neuroplasticity in selective cortical networks closely linked to its pathogenesis. The use of rTMS in chronic tinnitus is further supported by studies showing that rTMS has beneficial effects in a variety of neuropsychiatric disorders associated with focal hyperexcitability such as focal dystonias, focal epilepsy and posttraumatic stress disorder [11,21,22].

Taken together, a multitude of studies indicate a potential of low-frequency rTMS for the treatment of auditory phantom perceptions. With regard to chronic tinnitus, especially imaging guided rTMS has been proven to be beneficial in the treatment of chronic tinnitus. Since neuronavigated rTMS is sophisticated, costly and time consuming this kind of application is strongly limited and not applicable in routine activity. Due to these considerations, a simple and reliable method for stimulating the auditory cortex based on the 10–20 EEG system has been recently developed [23] and will be used in the present trial. Furthermore, studies so far investigated only relatively small populations. Therefore, in order to render rTMS as an effective treatment according to the principle of evidence based medicine, it is necessary to assess the efficacy of this treatment in a properly designed and conducted randomized-controlled trial in a large patient sample.

## Methods

### Design of the trial

**Figure 1:**
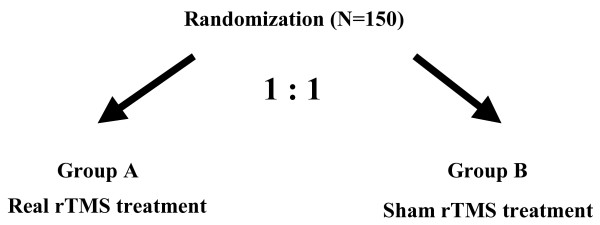
Trial design.

This is a multicenter, randomized, patient and observer-blind, sham-controlled, parallel-group study of 2 weeks 1 Hz rTMS treatment plus 24 weeks follow-up in patients with moderate to severe chronic tinnitus. Eligible patients will be randomly assigned to either 2 weeks real rTMS treatment or sham treatment (figure 1).

### Study population

Female and male subjects aged 18 to 70 years are eligible for study participation if they meet the following criteria:

#### Inclusion criteria

• Written informed consent

• Diagnosis of chronic tinnitus

• Patient has a score of ≥ 38 on the Tinnitus Handicap Inventory (assessed during routine clinical diagnostics in the last 12 weeks before start of treatment)

• Tinnitus duration of more than 6 months

• Age-adjusted normal sensorineural hearing determined by an audiogram within the last 12 weeks before start of treatment, i.e. no more than 5 dB below the 10 % percentile (DIN EN ISO 7029) of the appropriate age and gender group in all measured standard frequencies. Furthermore, no conductive hearing loss of more than 15 dB in neither of the measured standard frequencies.

• naïve regarding rTMS

#### Exclusion criteria

• Objective Tinnitus

• Other forms of tinnitus treatments at the same time

• Clinically relevant psychiatric co-morbidity as judged by an experienced psychiatrist, especially diagnose groups F1-3 according to the International Classification of diseases (ICD-10)

• Concomitant treatment with psychotropic drugs

• History or evidence of significant brain malformation or neoplasm, head injury, cerebral vascular events, neurodegenerative disorder affecting the brain or prior brain surgery

• Severe unstable somatic co-morbidity

• Cardiac pace makers, other electronic implants, intracranial metallic particles

• History of seizures or epileptic activity

• Pregnancy and lactation

• Women in child-bearing-age without contraception

• Patients who cannot communicate reliably with the investigator or who are not likely to cope with the requirements of the trial

• Participation in a clinical trial within the last 30 days before start of this clinical trial

### Participating centers

This study will be conducted by otorhinolaryngologists and psychiatrists with wide experience in the treatment of patients with chronic tinnitus from seven university hospitals and one municipal hospital all over Germany. All participating centers have experience in conducting rTMS-treatment studies.

### Study treatments

Screening is performed during the routine clinical tinnitus-consultations, and no additional study specific investigations are necessary to determine eligibility. After screening, written informed consent, baseline assessments, and randomization, patients will be treated with either real or sham low-frequency rTMS over a period of 2 weeks. rTMS will be administered according to current safety guidelines [24]. Magstim Super Rapid (The Magstim Company Ltd, Whitland, UK) or Medtronic MagPro (Medtronic GmbH & Co. KG, Düsseldorf, Germany) stimulators will be used for stimulation as available in the participating centers. Figure-of-eight-coils will be used for real stimulation. Sham stimulation will be carried out by tilting the coil 45° away from the skull with one wing touching the skull. The stimulation parameters have been chosen according to successful pilot studies [15,25] and modified with respect to duration, coil positioning and control condition. Recent data point to a dose-response relationship of rTMS treatment in a variety of neuropsychiatric diseases as well as in tinnitus treatment [23,26]. Furthermore, coil positioning using the 10–20 EEG-system has been proven to guarantee exact placement of the TMS coil over the auditory cortex without the need of using MR- and PET-guidance. Finally, finding an adequate placebo condition [27] is still under debate in clinical rTMS studies [28]. Here, sham stimulation will be performed by distortion of the magnetic coil 45° away from the skull with one wing touching the skull. In contrast to sham coils, this kind of sham stimulation does not induce a sufficiently strong magnetic field in the brain tissue to evoke any biological response but is still eliciting some kind of skull sensation. Especially in the context of tinnitus treatment studies, controlling for somatosensory stimulation seems to be critical since somatosensory input is able to modulate tinnitus sensation [1].

After screening, written informed consent, and baseline assessments, patients will be randomized to one of two parallel groups with the following stimulation parameters:

**Treatment group **will receive real stimulation: 2 × 5 sessions, 1 Hz rTMS, stimulation intensity 110% related to the individual resting motor threshold, 2000 stimuli per session, coil position 10–20 guided over left primary auditory cortex.

**Control group **will receive sham stimulation by distortion of the magnetic coil 45° away from the skull with one wing touching the skull. Coil positioning and stimulation parameters as in the treatment group.

At the beginning of the first treatment session, the individual resting motor threshold for the right abductor pollicis brevis muscle of each participant will be determined according to [29].

### Blinding

As already mentioned, blinding constitutes a substantial methodological challenge in rTMS studies. Therefore, a blinded design will be applied, in which patients and clinical raters will be blind to treatment conditions (observer blind). rTMS treatment will be provided by the non-blinded study staff. Patients have to be naïve regarding rTMS treatment and will not be informed about technical details of rTMS application. As the non-blinded study staff applying rTMS treatment needs to know whether real or sham rTMS is to be applied, and automatic rTMS is methodologically not feasible in clinical settings to date, the non-blinded study staff applying rTMS will be instructed not to communicate the treatment arm to anybody involved in patient management, in order to prevent any impact on blinding. The investigators performing all other assessments will be experienced physicians, not involved in rTMS treatment of the patient, and will perform all study specific procedures, e.g. getting informed consent, handing out clinical questionnaires or discussing any details about the conduct of the study with the patients. To assess successful blinding of the trial, each patient will be asked at the final visit (V14, day 181) whether or not he or she guesses to have been treated with real or sham rTMS.

### Objectives

#### General objective

Aim of this trial is to evaluate the efficacy of low-frequency rTMS in the treatment of chronic tinnitus. Based on the findings in pilot studies we hypothesize that two weeks treatment of low-frequency rTMS is more efficient in alleviating symptoms of chronic tinnitus and improving secondary symptoms like quality of life, cognitive functioning or depressive symptoms than sham treatment.

#### Primary objective

To evaluate the efficacy of real rTMS versus sham rTMS in the treatment of chronic tinnitus by means of a change of tinnitus severity according to the tinnitus questionnaire of Goebel & Hiller ([30], a self-rating questionnaire; baseline versus day 12).

#### Secondary objectives

To evaluate the efficacy of real rTMS versus sham rTMS

- in reducing tinnitus severity according to the tinnitus questionnaire of Goebel & Hiller and Tinnitus Handicap Inventory (THI; a self-rating questionnaire; [31]) during the follow-up period (screening versus baseline versus days 5, 12, 18, and weeks 10 and 26),

- in changes of quality of life of patients as measured by the SF 12 (a self-rating questionnaire [32]; baseline versus days 5, 12, 18, and weeks 10 and 26),

- in changes of depressive symptoms as measured by the Beck Depression Inventory (BDI; a self-rating questionnaire; [33]; baseline versus days 5, 12, 18, and weeks 10 and 26),

- in changes of psychometric parameters of tinnitus (tinnitus minimal masking level and tinnitus loudness) as assessed by audiological evaluation (screening versus day 18),

- in changes of structural and functional neuroplastic adaption processes as detected by voxel-based morphometry (VBM) and paired-pulse TMS (baseline versus day 12).

### Adjunctive measures

Further important factors that may influence treatment outcome may be clinical characteristics [34], the individual psychic resilience and personality traits. These will be assessed by using the German versions of the Tinnitus Sample Case History Questionnaire [[35]; please see also the URL in the Availability and requirments section , the NEO-FFI (a validated self-report questionnaire for measurement of the Five Factor model basic personality traits, neuroticism, extraversion, openness to experience, agreeableness, and conscientiousness; [36]) and the RS-11 (a patient self-rating to determine psychic resilience [37]).

### Enhancing quality of outcome measures

Tinnitus is a subjective sensation that cannot be measured by any objective parameter. Therefore, validated self rating scales (Tinnitus Handycap Inventory (THI) [31] and Tinnitus Questionnaire of Goebel and Hiller (TQ) [30]) are the only instruments to assess changes in tinnitus symptoms. Furthermore, tinnitus symptoms are often fluctuating and are influenced by attention. To minimize biases by spontaneous fluctuations or interactions with the trial staff, the rating scales will be handed out to the patient at the beginning of each visit *before *any other action will be taken. The patient should be seated in a quiet, comfortable room without any disturbances while answering the questionnaires. Furthermore, to increase the stability of the baseline measurement, tinnitus specific assessments (THI and TQ) will be performed three times (at the baseline visit and two times during the baseline week before the first treatment session), and the average over three measurements will be used as baseline value.

### Determination of sample size

Based upon our clinical experience, a difference of 5 points in tinnitus score (range: 0 – 80) according to the TQ is both clinically relevant and observable in a study setting [34], and 0.486 is a conservative estimate of the effect size [15,23,25,34]. Requiring a power (1-β) of 0.80 for detection of such an effect with a two-sided unpaired t-test (at a level of α = 0.05) leads to a sample size of 68 per arm (active vs. sham). A simulation study was conducted based on the pilot data, to study possible implications of incorporating center effects and baseline levels in the analysis, in which it was confirmed that the planned analysis of covariance indeed provided a power of about 0.80, given a sample size of 136. To account for the possibility of a substantially larger effect size, a scheduled interim-analysis will be carried out at the 50% recruitment level, so that a 138 subjects are needed in total (see e.g. [38]). Assuming that less than 5 percent of patients will fail to provide post-baseline data, 150 patients will be randomized to make sure that the target number of 138 evaluable patients will be reached.

### Randomization

Before the trial starts, randomization lists will be generated by the Clinical Trials Center, University Medical Center Freiburg, with a 1:1 treatment ratio, stratified by study center and with randomly varying block sizes. At each center, local investigators will have to confirm eligibility of enrolled patients and the non-blinded study staff will forward their request for randomization by fax to the randomization center, located at the department of the lead investigator. There, the randomization lists will be securely stored with none of the clinical staff involved in the trial having access to it. The randomization center will send the allocated treatment by fax to the local non-blinded staff performing rTMS. In addition, a standard email including a statement that the patient has been successfully randomized, will be send to both, the blinded and non-blinded study staff of the center as well as to the monitor.

### Statistical Analysis

The primary endpoint for evaluation of efficacy of rTMS for tinnitus is the change from baseline to day 12 in the tinnitus score (TQ total score). The treatment effect will be estimated on an intention-to-treat basis including all patients with post-baseline data (carrying forward day 5 measurements if day 12 is missing), in an analysis of covariance including treatment and average baseline score as fixed factors and center as a random factor. Interim analysis for TQ change at day 12 will be carried out on the first 76 randomized patients following an O'Brien and Fleming type sequential design with overall two-sided significance level 0.05 (0.0052 at interim, 0.0481 at final analysis; [38]). If more than 5% of patients fail to provide post-baseline data, the primary analysis will be complemented by sensitivity analyses exploring potential biases induced by patterns of missingness which will be investigated by logistic regression on comprehensive baseline information.

Secondary analyses of TQ change over time will be performed in longitudinal linear mixed models based with a pre-specified model-building strategy. For the other secondary objectives with respect to rating scales (THI, SF 12, BDI, and CGI) a similar strategy will be employed as applicable. For changes in cortical excitability and changes in psychometric parameters of tinnitus assessed by audiological examination, one-way ANOVA analysis will be used, and for changes in brain structure voxel-based morphometry (VBM) will be used.

### Procedures

#### Visit schedule

An overview on the time schedule for assessing and recording of efficacy and safety parameters is given in table 1. Baseline assessments are followed by the two weeks treatment period, when the patient is seen daily from Monday to Friday (total of 10 visits). During the follow-up, patients will be seen at day 18 (visit 12), day 67 (visit 13) and at the final visit at day 181. In case of treatment discontinuation, efficacy parameters should be assessed for the whole study period at all outstanding visits (visits 6, 11–14, respectively). For patients who discontinue the study, the premature termination visit will be performed whenever possible.

**Table 1:** Study Plan

	**Screening**	**Baseline**	**R**^3^	**Treatment**										**Follow-Up**		
Visits	V0	V1		V2	V3	V4	V5	V6	V7	V8	V9	V10	V11	V12	V13	final visit^4^

**Week**		Week 0		Week 1					Week 2					Week 3	Week 10	Week 26/PTV

**Day**		- 11 to -9		1	2	3	4	5	8	9	10	11	12	17 – 19	66–68	180 – 182
**Rating Scales (THI)**	X^1^	X^2^						X					X	X	X	X
**Otologic examination and audiological assessment**	X^1^													X		
**Informed Consent**	X															
**Demographic Data**	X															
**Medical History**	X															
**Documentation of Comorbidity**	X	X														
**Documentation of concomitant medication**	X	X											X	X	X	X
**Physical examination**		X											X			X
**Vital Signs**		X		X									X			X
**Rating Scales (Goebel&Hiller, BDI, SF 12)**		X^2^						X					X	X	X	X
**Tinnitus-Severity**		X		X				X					X	X	X	X
**NEO FFI, RS-11**		X														
**CGI**		X		X				X					X	X	X	X
**Neuropsychological Assessment**		X											X			
**Cortical Excitability (pTMS)**		X											X			
**Structural Neuroimaging (VBM)**		X											X			
**Inclusion/Exclusion**		X														
**Randomization**			X													
**rTMS Treatment**				X	X	X	X	X	X	X	X	X	X			
**Documentation of Adverse Events**				X	X	X	X	X	X	X	X	X	X			

^1 ^= results of routine clinical investigations

^2 ^= each patient receives at V1 two versions of THI and TQ to be completed on Monday and Friday before V2

^3 ^= randomisation

^4 ^= Visit 14 or PTV = Premature Termination Visit

#### Data collection

All randomized patients will receive a profound physical, neuropsychological and audiological examination at the screening and baseline visits, respectively. Beside a detailed physical examination, vital signs (e.g. heart rate, blood pressure, height and weight) will be determined at visits 2, 11 and 14. Neuropsychological parameters (test for attentional performance; TAP) will serve as a safety criterion to detect any deterioration in cognitive function and will be repeated at visit 11 (day 12) at the end of the treatment period. Audiological examinations at screening and visit 12 (day 18) will serve as both secondary outcome (tinnitus masking and loudness matching) and safety parameter (pure tone audiometry). Concomitant medication will be documented at screening and baseline, visit 11 at the end of the treatment period as well as at each follow-up visit. Tinnitus-specific rating scales (THI and TQ) will be performed three times during the baseline week before treatment and thereafter at visits 6 and 11 – 14 together with BDI and SF-12. Moreover, information on any adverse event, including side effects of study treatments, will be collected during the treatment period (visits 2 – 11). An independent data safety and monitor board (DSMB) consisting of clinical and statistical experts will critically assess adherence to the study protocol as well as to ICH-GCP-guidelines. Together with the sponsor, the DSMB will decide whether the trial has to be terminated prematurely. Possible reasons encompass e.g. safety concerns, unexpected high discontinuation rates or a large effect size on TQ at day 12 determined by the interim analysis.

### Timing

The start of the study is February 2008. With a planned number of 138 patients providing post-baseline data, a drop out rate for the primary outcome of less than 5% and an expected screening to inclusion ratio of 5:1, 750 patients will have to be assessed for eligibility in all 8 centers, and 150 will be allocated for trial participation. The recruitment and treatment period will be 27 months, with 6 additional months to complete follow-up. Analyses and reporting will be estimated to be 6 months, resulting in a total duration of about 3 years.

### Ethical aspects

#### Safety of rTMS

Applied under previously published safety guidelines [24], rTMS has been proven to be a safe and well tolerated therapy in a broad range of studies encompassing distinct neuropsychiatric diseases. Considerable side effects of rTMS encompass potentially occurring physical discomfort on the scalp during stimulation and headache after rTMS with an incidence of about 5%. Other relevant side effects are not expected if known contraindications (see exclusion criteria) are respected.

#### Ethics committee

The study protocol as well as the documents to obtain patient's informed consent have been approved by the independent ethics committee of the coordinating center at the university of Regensburg on October 24^th ^2006. The study will be performed in accordance with the ethical principles that have their origin in the Declaration of Helsinki and are consistent with the ICH guidelines for Good Clinical Practice. The trial has been registered at the International Standard Randomized Controlled Trial Number Registration (ISRCTN89848288).

#### Patient information and informed consent

The patient will be fully informed about the nature, purpose, possible risks and benefit of the study by the investigator before any study specific actions will take place. Patients will be notified that they are free to discontinue from the study at any time without any disadvantages for further clinical care. There will be sufficient time to ask questions and to consider the information provided. If the patient is willing to participate in the trial, he or she will be asked to sign the informed consent form. Patients must be withdrawn from study treatment when judged necessary by the investigator. Reasons may be for example safety concerns, severe non-compliance, incorrect enrolment or randomization. Follow-up will end in case of withdrawal of the informed consent by the patient.

#### Data management

Data management will be performed by the Center for Clinical Studies, University of Regensburg. An Access database will be used and after closure of the database, data will be transferred to the Clinical Trials Center, University Medical Center Freiburg for data analysis.

#### Monitoring

Monitoring will be performed according to current ICH-GCP-guidelines. Each center will be initiated by a standardized conference call using a presentation describing the basic principles of the study and most relevant procedures (e.g. getting informed consent, randomization, treatment procedures, emergency unblinding). In addition, each center will be visited regularly by the monitor depending on the patient recruitment.

## Discussion

Although chronic tinnitus is a frequent and often disabling condition, no treatment can yet be considered well established in terms of providing replicable long-term reduction of tinnitus impact in excess of placebo effects [39]. Therefore, new therapeutic approaches are highly needed. Based on recent advances in the understanding of the pathophysiology of tinnitus and based on an increasing amount of promising pilot data, rTMS seems to hold high potential as a new treatment tool. However, clinical data on the efficacy are limited, mainly due to the small sample sizes of available studies. Several methodological difficulties had to be considered in the design of this trial. First tinnitus is a purely subjective phenomenon, which is difficult to assess. Second, only recently an expert consensus for treatment outcome measurements in tinnitus patients has been published [35]. Third the blinding condition is more difficult to perform in rTMS studies than in pharmacologic studies and fourth there is only very limited experience with multi-center placebo-controlled trials conducted according to ICH-GCP guidelines for both the indication tinnitus and the intervention rTMS.

Here we present a study protocol for a multi-center, placebo-controlled clinical rTMS trial conducted according to ICH-GCP guidelines and to the above mentioned expert consensus [35] and involving professional monitoring. The presented study protocol takes into account all the described methodological difficulties and demonstrates the feasibility of a controlled multi-center rTMS trial thereby enhancing the quality of the collected data. The study sample is one of the largest ever investigated in TMS studies and the largest sample of patients with chronic tinnitus.

## Abbreviations

BDI: Beck Depression Inventory; DSMB: Data Safety Monitor Board; GCP: Good Clinical Practice; ICH: International Conference on Harmonization; LOCF: last observation carried forward; MR: magnet resonance tomography; NEO FFI, NEO: Five Factor Inventary; RTMS: repetitive transcranial magnetic stimulation; THI: Tinnitus Handicap Inventory; TQ: Tinnitus Questionnaire of Goebel&Hiller; VBM: Voxel-based Morphometry; RS-11: short for of the German questionnaire to assess resilience; SF 12: Quality of life assessment scale.

## Availability and requirements

Tinnitus Sample Case History Questionnaire (German, English, French, Italian, Spanish, Dutch, Czech, Portugese):



## Competing interests

The author(s) declare that they have no competing interests.

## Authors' contributions

ML, MK, TK, PE, GH and BL contributed to the design of the study. The study protocol has been written by ML, PE and BL, the manuscript has been drafted by ML and BL. Responsible for recruitment and follow-up of the enrolled patients as well as coordination of the multi-center trial will be ML and BL. The statistical design and all statistical analyses will be performed by HB and EG.

## Monitoring

Dr. J. Reisinger, multi-service-monitoring, Regensburg, Germany

## Trial coordinating center

University of Regensburg, Prof. Dr. G. Hajak (Principal Investigator), Department of Psychiatry. Center for Clinical Studies of the University of Regensburg, Prof. Dr. M. Koller (data management).

## Pre-publication history

The pre-publication history for this paper can be accessed here:


